# Supplementary material for: Evaluation of an on-site sanitation intervention against childhood diarrhea and acute respiratory infection 1 to 3.5 years after implementation: Extended follow-up of a cluster-randomized controlled trial in rural Bangladesh
Source: PLoS Med. 2022 Aug 8;19(8):e1004041. doi: 10.1371/journal.pmed.1004041 (PMC9394830; doi:10.1371/journal.pmed.1004041)
Supplement: S1 CONSORT Checklist — (PDF) [file pmed.1004041.s001.pdf]

Table S1: CONSORT 2010 checklist of information to include when reporting a randomized trial

Section/Topic	Item No	Checklist item	Reported on page No
**Title and abstract**			
	1a	Identification as a randomised trial in the title	Title
	1b	Structured summary of trial design, methods, results, and conclusions (for specific guidance see CONSORT for abstracts)	Abstract
**Introduction**			
Background and objectives	2a	Scientific background and explanation of rationale	Introduction
	2b	Specific objectives or hypotheses	Introduction, paragraph 5
**Methods**			
Trial design	3a	Description of trial design (such as parallel, factorial) including allocation ratio	Methods: Randomization and Masking, Interventions, Substudy Design
	3b	Important changes to methods after trial commencement (such as eligibility criteria), with reasons	NA
Participants	4a	Eligibility criteria for participants	Methods: Randomization and Masking, Substudy Design
	4b	Settings and locations where the data were collected	Methods: Randomization and Masking
Interventions	5	The interventions for each group with sufficient details to allow replication, including how and when they were actually administered	Methods: Interventions
Outcomes	6a	Completely defined pre-specified primary and secondary outcome measures, including how and when they were assessed	Methods: Study Outcomes
	6b	Any changes to trial outcomes after the trial commenced, with reasons	NA
Sample size	7a	How sample size was determined	Methods: Statistical Analysis, second paragraph
	7b	When applicable, explanation of any interim analyses and stopping guidelines	NA
Randomisation:			
Sequence generation	8a	Method used to generate the random allocation sequence	Methods: Randomization and Masking
	8b	Type of randomisation; details of any restriction (such as blocking and block size)	Methods: Randomization and Masking

Allocation concealment mechanism	9	Mechanism used to implement the random allocation sequence (such as sequentially numbered containers), describing any steps taken to conceal the sequence until interventions were assigned	Methods: Randomization and Masking
Implementation	10	Who generated the random allocation sequence, who enrolled participants, and who assigned participants to interventions	Methods: Randomization and Masking
Blinding	11a	If done, who was blinded after assignment to interventions (for example, participants, care providers, those assessing outcomes) and how	Methods: Randomization and Masking
	11b	If relevant, description of the similarity of interventions	NA
Statistical methods	12a	Statistical methods used to compare groups for primary and secondary outcomes	Methods: Statistical Analysis
	12b	Methods for additional analyses, such as subgroup analyses and adjusted analyses	Methods: Effect Modification
**Results**			
Participant flow (a diagram is strongly recommended)	13a	For each group, the numbers of participants who were randomly assigned, received intended treatment, and were analysed for the primary outcome	Figure S1
	13b	For each group, losses and exclusions after randomisation, together with reasons	Figure S1
Recruitment	14a	Dates defining the periods of recruitment and follow-up	Results, second paragraph
	14b	Why the trial ended or was stopped	NA
Baseline data	15	A table showing baseline demographic and clinical characteristics for each group	Previously published (described in text under Results, first paragraph)
Numbers analysed	16	For each group, number of participants (denominator) included in each analysis and whether the analysis was by original assigned groups	Results, first paragraph; Results: Intervention Effects
Outcomes and estimation	17a	For each primary and secondary outcome, results for each group, and the estimated effect size and its precision (such as 95% confidence interval)	Results: Intervention Effects; Table 1
	17b	For binary outcomes, presentation of both absolute and relative effect sizes is recommended	Results: Intervention Effects; Table 1
Ancillary analyses	18	Results of any other analyses performed, including subgroup analyses and adjusted analyses, distinguishing pre-specified from exploratory	Results: Effect Modification; Table 2
Harms	19	All important harms or unintended effects in each group (for specific guidance see CONSORT for harms)	NA
**Discussion**			
Limitations	20	Trial limitations, addressing sources of potential bias, imprecision, and, if relevant, multiplicity of analyses	Discussion, fifth and sixth paragraphs
Generalisability	21	Generalisability (external validity, applicability) of the trial findings	Discussion, second, fourth, and fifth paragraphs
Interpretation	22	Interpretation consistent with results, balancing benefits and harms, and considering other relevant evidence	Discussion

**Other information**			
Registration	23	Registration number and name of trial registry	Abstract; Methods: Randomization and Masking
Protocol	24	Where the full trial protocol can be accessed, if available	Methods: Study Outcomes
Funding	25	Sources of funding and other support (such as supply of drugs), role of funders	Funding
