# Supplementary material for: Evaluation of an on-site sanitation intervention against childhood diarrhea and acute respiratory infection 1 to 3.5 years after implementation: Extended follow-up of a cluster-randomized controlled trial in rural Bangladesh
Source: PLoS Med. 2022 Aug 8;19(8):e1004041. doi: 10.1371/journal.pmed.1004041 (PMC9394830; doi:10.1371/journal.pmed.1004041)
Supplement: S1 Fig — Parent trial data includes substudy participants and those not enrolled in the substudy. Observations are separated by approximate follow-up time after the sanitation intervention was implemented in participating households. (PDF) [file pmed.1004041.s002.pdf]

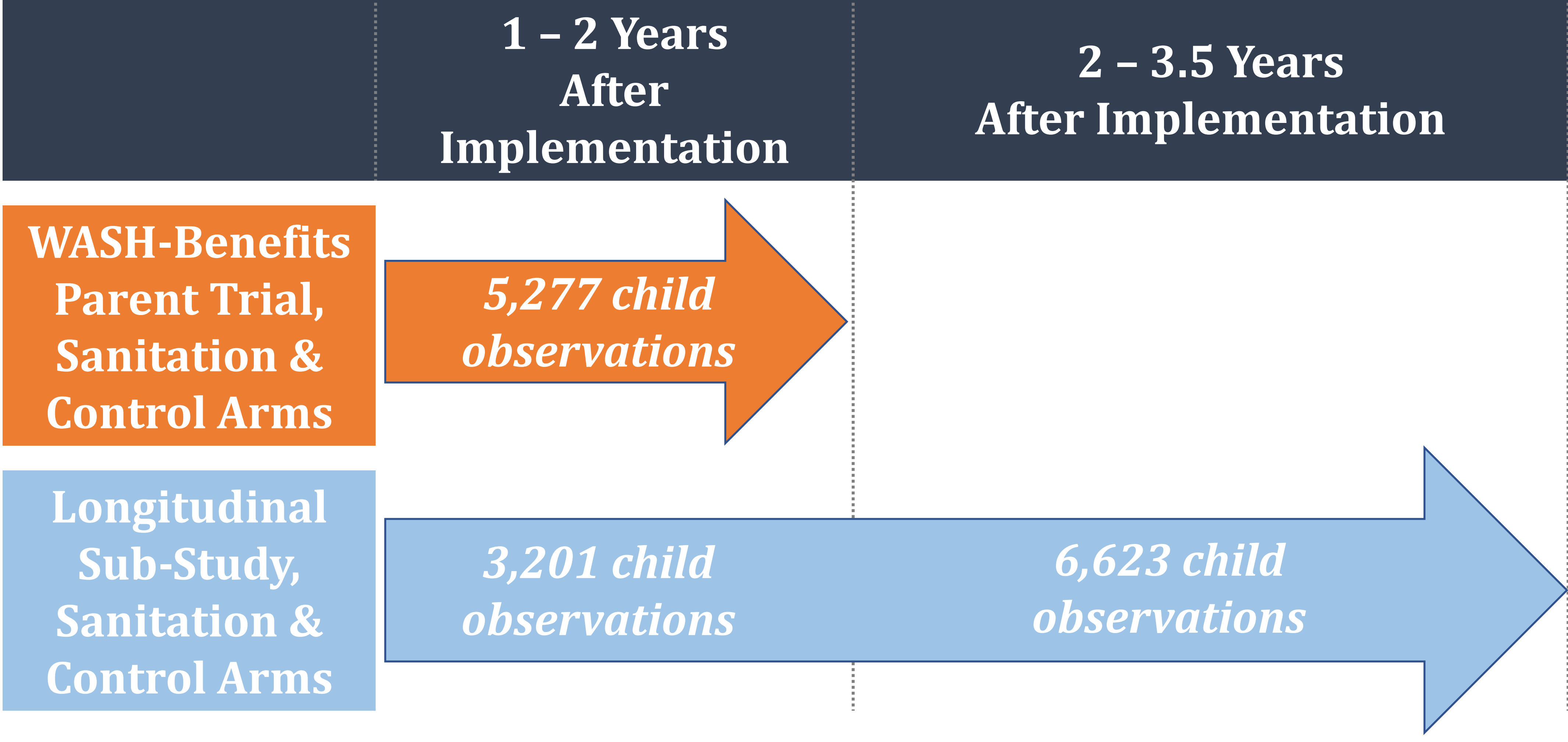
1 – 2 Years After Implementation
2 – 3.5 Years After Implementation
WASH-Benefits Parent Trial, Sanitation & Control Arms
5,277 child observations
Longitudinal Sub-Study, Sanitation & Control Arms
3,201 child observations
6,623 child observations
