# Supplementary material for: Evaluation of an on-site sanitation intervention against childhood diarrhea and acute respiratory infection 1 to 3.5 years after implementation: Extended follow-up of a cluster-randomized controlled trial in rural Bangladesh
Source: PLoS Med. 2022 Aug 8;19(8):e1004041. doi: 10.1371/journal.pmed.1004041 (PMC9394830; doi:10.1371/journal.pmed.1004041)
Supplement: S3 Fig — Prevalence estimates shown separately for (i) data from parent trial for all controls in the parent trial; (ii) data from parent trial for the subset of controls that also participated in this substudy; and (iii) data from substudy for controls in this substudy. Shaded bands represent 95% credible intervals around each prevalence estimate. Monsoon and dry seasons are indicated with vertical dashed lines. (PDF) [file pmed.1004041.s004.pdf]

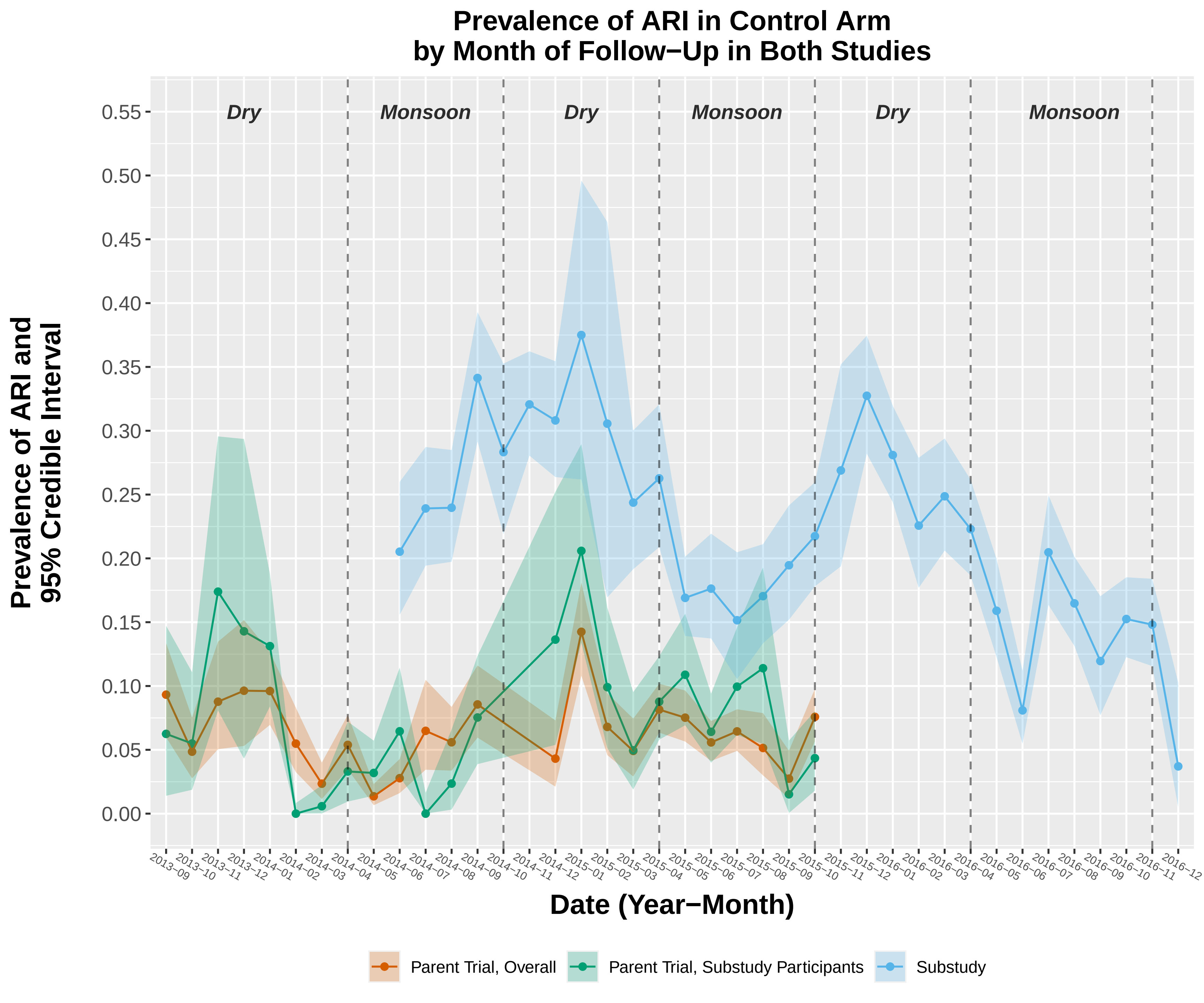

Prevalence of ARI in Control Arm
by Month of Follow−Up in Both Studies
Dry
Monsoon
Dry
Monsoon
Dry
Monsoon
Prevalence of ARI and
95% Credible Interval
0.00
0.05
0.10
0.15
0.20
0.25
0.30
0.35
0.40
0.45
0.50
0.55
Date (Year−Month)
Parent Trial, Overall
Parent Trial, Substudy Participants
Substudy
