# Supplementary material for: Evaluation of an on-site sanitation intervention against childhood diarrhea and acute respiratory infection 1 to 3.5 years after implementation: Extended follow-up of a cluster-randomized controlled trial in rural Bangladesh
Source: PLoS Med. 2022 Aug 8;19(8):e1004041. doi: 10.1371/journal.pmed.1004041 (PMC9394830; doi:10.1371/journal.pmed.1004041)
Supplement: S4 Fig — Prevalence estimates shown separately for (i) data from parent trial for all controls in the parent trial, (ii) data from parent trial for the subset of controls that also participated in this substudy; and (iii) data from substudy for controls in this substudy. Shaded bands represent 95% credible intervals around each prevalence estimate. Monsoon and dry seasons are indicated with vertical dashed lines. (PDF) [file pmed.1004041.s005.pdf]

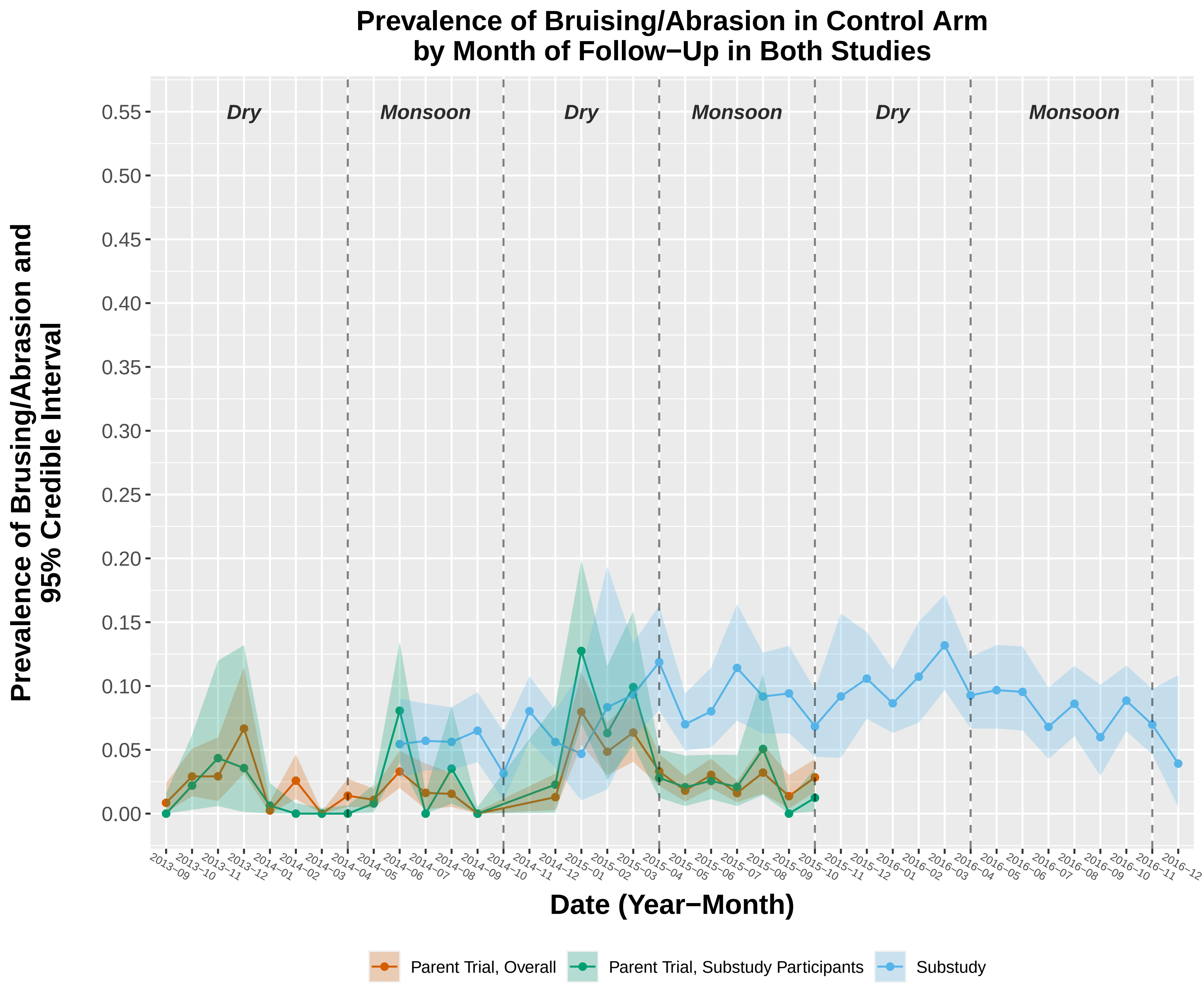

Prevalence of Bruising/Abrasion in Control Arm
by Month of Follow−Up in Both Studies
Prevalence of Brusing/Abrasion and
95% Credible Interval
Dry
Monsoon
Dry
Monsoon
Dry
Monsoon
0.55
0.50
0.45
0.40
0.35
0.30
0.25
0.20
0.15
0.10
0.05
0.00
2013−09
2013−10
2013−11
2013−12
2014−01
2014−02
2014−03
2014−04
2014−05
2014−06
2014−07
2014−08
2014−09
2014−10
2014−11
2014−12
2015−01
2015−02
2015−03
2015−04
2015−05
2015−06
2015−07
2015−08
2015−09
2015−10
2015−11
2015−12
2016−01
2016−02
2016−03
2016−04
2016−05
2016−06
2016−07
2016−08
2016−09
2016−10
2016−11
2016−12
Date (Year−Month)
Parent Trial, Overall
Parent Trial, Substudy Participants
Substudy
