# Supplementary material for: Evaluation of an on-site sanitation intervention against childhood diarrhea and acute respiratory infection 1 to 3.5 years after implementation: Extended follow-up of a cluster-randomized controlled trial in rural Bangladesh
Source: PLoS Med. 2022 Aug 8;19(8):e1004041. doi: 10.1371/journal.pmed.1004041 (PMC9394830; doi:10.1371/journal.pmed.1004041)
Supplement: Attachment — Submitted filename: WASHB-longitudinal_child_health_response_Apr-14-2022.docx [file pmed.1004041.s006.docx]

**Evaluation of an on-site sanitation intervention against childhood diarrhea and acute respiratory infection 1 to 3.5 years after implementation: Extended follow-up of a cluster randomized controlled trial in rural Bangladesh**

***** Editor:**

Please adapt the title to better reflect journal style, and we suggest: "Evaluation of an on-site sanitation intervention against child diarrhea and acute respiratory infection outcomes 1 to 3.5 years after implementation in rural Bangladesh: Extended follow-up of a cluster-randomized controlled trial".

Changed to “Evaluation of an on-site sanitation intervention against childhood diarrhea and acute respiratory infection 1 to 3.5 years after implementation: Extended follow-up of a cluster randomized controlled trial in rural Bangladesh”

You use the descriptor "substudy" in the text, and as an alternative this could be included in the title for consistency.

“Substudy” is useful to put our results in context within a broader trial, but our study is also able to stand alone and used different data. We feel that adding “substudy” to the title would muddy the true nature of the analysis.

In the abstract and throughout the paper, please quote p values alongside 95% CI, where available.

Added.

Please add a new final sentence to the "Methods and findings" subsection of the abstract, which should begin "Study limitations include ..." or similar and should quote 2-3 of the study's main limitations.

Added: “Study limitations include use of caregiver-reported symptoms to define health outcomes and limited data collected after promotion ceased.”

At line 60 (abstract) and at other points in the paper, please soften the language to reflect the research design and the observed changes. We suggest amending the text to "The apparent benefit of the WASH ...".

We attempted to differentiate between “effects” as a take-home message from this work and “effects” as a statistical term referring to differences in outcomes between randomly assigned intervention and control arms.

In the abstract, as you suggested, and in the final paragraph of the Discussion, we used “effect” as a broad take-home message. To temper the interpretation, we added “observed” in both places, as suggested.

Elsewhere, we use “effect” or “effects” to describe differences in outcomes between randomly assigned study arms. Although there is always risk of bias in epidemiological studies, we believe that our results can accurately be labeled as causal intervention effects for the following reasons. The interventions had high levels of adherence in the intervention group so intention-to-treat analysis will not underestimate true causal effects. Our results are unlikely to be affected by confounding, due to randomization and good covariate balance between intervention arms. Selection bias is unlikely as we had low loss to follow-up and household that left vs. remained in the study were similar in their characteristics. Differential measurement bias due to the non-blinded intervention and subjectively reported outcomes is possible. We thoroughly discuss the potential for measurement bias affecting our results in the Discussion and highlight some additional information that supports interpreting our results as true causal effects, such as comparable intervention effects on objectively measured enteric infections and the biological plausibility of the observed stronger intervention effects during the rainy season.

Thus, we maintained the use of “effect(s)” in the rest of the manuscript when referring to differences in outcomes between arms. We are happy to temper any specific instances of broader conclusions if we missed any.

After the abstract, please add a new and accessible "Author summary" section in non-identical prose. You may find it helpful to consult one or two recent research papers in PLOS Medicine to get an impression of the preferred style.

Added an author summary after the Abstract.

Thank you for providing information about your analysis plan. Please highlight any analyses that were not prespecified.

All analyses were pre-specified.

At line 130, please adapt the text to "We aimed to ..." or "We assessed ...".

Changed to “We assessed…”

In the Methods section, it may be worthwhile subdividing the subsection on "Statistical analysis" into two subsections on "Study procedures" and "Statistical analysis" or similar.

Divided into “Study Outcomes” and “Statistical Analysis”.

Please remove the "Role of the funding source" statement at the end of the Methods section.

Removed.

Again at line 295, please temper the language and quote, alongside the apparent reduction of 19%, the point estimate and 95% CI.

Added point estimate and 95% CI.

At line 295 and elsewhere, please use the style "... under 5 years".

Changed each instance of “under five” or “under three” to “under 5 years” or “under 3 years”.

Please adapt reference call-outs to the following style throughout the paper: "... in children [1,2]." (noting the absence of spaces within the square brackets).

Reformatted to PLOS Medicine style available in Mendeley.

Please remove the information on funding, competing interests and data sharing from the end of the main text. In the event of publication, this information will appear in the article metadata, via entries in the submission form.

Removed.

In the reference list, please convert all italics and boldface to plain text. Where appropriate, please list 6 author names rather than 3, followed by "et al.".

Reformatted to PLOS Medicine style available in Mendeley.

Is reference 27 a paper in preparation? If so, please supply it with your revision for the reviewers to take into account, or make it available as a preprint.

The referenced paper is currently under review. We will submit a copy along with these revisions.

Thank you for providing a completed CONSORT checklist. Please adapt this so that individual items are referred to by section (e.g., "Methods") and paragraph numbers, not by page or line numbers as these generally change in the event of publication.

CONSORT checklist was adapted. The following was added to Methods under *Substudy Design*: “We followed CONSORT guidelines in reporting the design and results of this trial (S1 Table).”

Please break the checklist out into an individual file, labelled "S1_CONSORT_Checklist" or similar and referred to as such in the Methods section (main text).

Will resubmit as individual file.

***** Reviewer #1:**

Statistical review

This paper reports a long-term follow-up of a substudy from a cluster randomised trial investigating a sanitation intervention.

Generally the study was written and reported well. I have some minor comments:

1.      Abstract: "There were no differences in intervention effects between periods with high-intensity vs. low-intensity/no promotion." - I would add 'significant' here to indicate this was formally tested.

Added.

2.      Substudy Design: Was there a statistical justification for the substudy sample size? I would add a brief note on the justification whether it was statistical or based on feasibility (I note later that the authors show the effect size the substudy has 80% power for).

We clarified in the text that the sample size was based on power calculations for a different analysis (new text in bold):

“**The sample size of this substudy was based on power calculations for a separate analysis of fecal contamination.** Before this analysis, we estimated minimum detectable effects for diarrheal disease and ARI, **given the existing sample size and** the observed prevalence values in the control arm of the substudy.”

3.      Statistical analysis: can the authors comment on missing data and how this was accounted for (presumably the analysis model is valid under a MAR assumption).

In the first paragraph under Results we describe loss to follow-up for the two study arms: “Eighty percent of households provided data for all eight survey rounds, and 96% completed at least six surveys. Loss to follow-up was similar between arms, and participants that left vs. completed the study were similar in their characteristics.[28]” Therefore, missing data due to loss to follow-up do appear to follow a MAR assumption. In our previous publication using data from this substudy, intervention effects on environmental contamination did not change when we excluded data from households that did not participate in all eight sampling rounds.

In addition, we added the following in the third paragraph under Results to ensure we mention additional missing data due to incomplete questionnaire answers: “Fewer than 1% of observations were missing health outcome data and were not included in this analysis.”

4.      Although not strictly significant, the negative control item appears marginally significant from the CI - is this concerning to the authors about the interpretation of the primary outcome? This is briefly remarked upon in the discussion so may need nothing additional adding.

We agree that this is an important result despite not being significant at .05. The implication could be that our primary results are the result of information bias, which we discuss in the Discussion, including the implication of our bruising result. We provide some specific reasons that support causal interpretation of our diarrhea results, based on the intervention’s effects on objectively measured enteric infections and the biologically plausible seasonal effects on diarrhea.

5.      Line 283-284: there is evidence the effect is modified by season, but the interaction pvalue for index household status isn't significant: so I'm not sure it's correct to say there was evidence of effect modification for index household status. Perhaps the authors could make clear the evidence for this is stronger for season than index household?

We added p-values to contextualize our overall results statement: “Intervention effects on diarrheal disease were modified by index household status (p = .15) and season (p = .02), but not by differing promotion intensity (p = .86)”.

We still state that index household status did modify effects because alpha values as high as .20 are conservatively used to detect effect modification, and because subgroup effects by index household status show qualitative differences (significant intervention effect for index households but no effect in other households).

***** Reviewer #2:**

The authors present an interesting sub-study of the WASH-B Bangladesh trial that aims to estimate the effect of the sanitation arm of the intervention over a longer time period (3.5 years) than the original trial. The authors rightly point out that this work has the potential to help fill a current gap in the literature: we know very little about the long-term sustainability of WASH intervention effects. However, the main analysis pools results from 1-3.5 years post intervention, a period that overlaps with the parent trial, leaving the reader to ponder whether the intervention effects reported here truly reflect long-term intervention effects or whether they are skewed by measurements taken 1-2 years post-intervention (a period during which an intervention effect has previously been reported). Further specific comments follow.

We thank the reviewer for their thoughtful and helpful comments. We note that we assessed temporal changes in intervention effects through our effect modification analysis, which we present as a comparison between periods of high vs. low/no behavioral promotion. We found no differences in intervention effects during high promotion (1-2 years after intervention implementation) vs. low/no promotion (2-3.5 years after intervention). Therefore, we conclude that the intervention’s effects persisted throughout the duration of the study, and we suggest that tapering behavioral promotion did not reduce the intervention’s effectiveness.

Abstract - 
The prevalence estimates - clarify that these are pooled estimates.

Added “Over the entire study period…” to indicate that the reported effects include all observations, in contrast to the effect modification results between periods with high- vs. low-intensity promotion/no promotion.

Methods

Intervention 
-       If possible, it would be useful to include an approximate number of visits/month during years - 2-3 when visits begin to taper off.

We updated the following sentence in Interventions under Methods (new text in bold):

“Promotion intensity gradually decreased between years 2-3**, beginning with one visit per month,** and all promotion activities stopped approximately three years after intervention initiation.”

-       Lines 166- 168: How long after the intervention was implemented was use/access of latrines and use of saniscoops/potties measured?

Use was measured throughout the substudy period. We updated the sentence to specify:

“Spot check observations and structured questionnaires demonstrated that the intervention increased access to and use of hygienic latrines throughout our study period (1-3.5 years after implementation), while uptake was low and inconsistent for sani-scoops and potties.[27]”

Statistical analysis

-       No adjustment for other covars (like child age) given good balance at baseline - but there would have been births into compounds/household and some attrition over the 2.5 year follow-up. How were changes in the population handled and could this bias results?

Attrition was similar between arms, and participants who left vs. remained in the study were similar in their characteristics. The rate of new births in the compound was similar between arms, with new births reported at 9% and 10% of visits in the sanitation and control arms, respectively. Therefore, the average age of children was similar in both study arms, and overall we have consistently found good balance between arms for all covariates measured.

Results

-       Lines 246 - 249: You switch between discussing enrolled households and descriptive statistics for compounds. It might be useful to clarify in the methods that there was one index household per compound (if this was the case).

We explain the distinction between households and compounds under *Interventions* in the Methods. We changed the first line of Results to “We enrolled 720 index households and their familial compounds”, in order to clarify again before reporting compound-level statistics.

Also, it might be useful to provide some sort of population normalised measure of number of latrines per compound (latrines/person or latrines/household) to better understanding sharing.

We added the following in the designated paragraph: “There were an average of 4.2 compound residents per latrine in the sanitation arm compared to 7.1 in the control arm”

-       Lines 250-252: Did you measure upgrades to latrines in control households over the same period?

No, this question was specific to the intervention activities.

-       Lines 257 - 262: Was there any differential movement in or out of the study population? i.e. losses to follow-up or differences in births/movement into study sites.

We added the following to the first Results paragraph: “Eighty percent of households provided data for all eight survey rounds, and 96% completed at least six surveys. Loss to follow-up was similar between arms, and participants that left vs. completed the study were similar in their characteristics.[28]”

We also added the following to the second paragraph on child observations: “The rate of new births in the compound was similar between arms, with new births reported at 9% and 10% of visits in the sanitation and control arms, respectively.”

-       Line 267: It would be useful to mention the follow-up rounds during which bruising/abrasion was highest and lowest as is done for diarrhea and ARI.

Changed sentence to: “The overall prevalence of bruising/abrasion was 8.2%; the prevalence was highest during the sixth round (10.6%) and lowest during the first round (5.7%).”

-       Intervention effects section: As the introduction speaks at length about the gap in the literature around measuring intervention effects over extended periods of times, it would seem relevant to include results that speak more directly to this gap, i.e. intervention effects over time/ at individual time points that extend past the original trial results. This will reduce sample size substantially but may still be informative.

We achieved this through our effect modification analysis between periods of high vs. low/no promotion. Since promotional activities were reduced over time, we could not separate time vs. promotion, and we elected to frame our analysis around the timing of promotion where the high promotion period corresponds to 1-2 years after intervention implementation and the low/no intervention period corresponds to 2-3.5 years after intervention implementation. However, the lack of effect modification over promotional periods also indicate that the intervention’s effects did not wane over the course of the study.

We added text under *Effect Modification* in Results to reiterate the connection between time and promotional intensity:

“[Intervention effects were not modified] by differing promotion intensity **over time (i.e., high-intensity promotion in years 1-2 after intervention implementation vs. low-intensity/no promotion in years 2-3.5)** (p = .86)”.

-       Line 283: Would clarify what "index house status" means as I had to go back through methods. Also, as intervention promotion was targeted at the index household, is it possible that promotion intensity would be more important in this targeted subgroup of the study population?

Changed sentence to: “Intervention effects on diarrheal disease were modified between children living in the index household (i.e., primary intervention recipients in the compound) vs. other households in the compound…”

It is possible that promotional intensity would be more important for index households, who were primarily targeted, although all households in the compound were invited to participate in promotional activities. We note this in the second paragraph in the Discussion. However, our analysis of effect modification by promotional intensity suggests that promotion may have been less important than the hardware upgrades that were also primarily targeted to index households.

Discussion:

-       Lines 306 - 309: Could the difference between index and non-index households be due to differences in the physical infrastructure provided to index vs non-index households rather than the difference in promotion? Especially since you see no effect modification by promotion intensity. Additionally, in lines 313-324 you suggest that it is infrastructure improvements that may be the reason for the reduction in diarrhea.

Yes, it is an important observation that should be noted. We added the following at the end of this paragraph.

“We note that at the baseline of the parent trial, roughly half of index households in the sanitation and control arms owned their own latrine, while those without their own latrine typically shared with another household in the compound. [16] Index households were the only households guaranteed a new improved latrine if they did not have one, while other latrines in the compound were eligible to be upgraded if necessary. Thus, all index households in the sanitation arm had their own latrine after the intervention was implemented, and increased personal access to an improved latrine may have led to the unique effects among index household children

-       Lines 314 - 315: In the results section you state that 97% control compounds and 100% intervention compounds had a latrine. Is this referring to the same statistic? If so, please clarify if you mean compound or household.

The numbers are different but match (only households that reported no latrine in the compound reported not using a latrine within the compound). The text has been edited to reflect this:

‘At the onset of this substudy, 97% of index households in the control arm reported using a latrine within their compound”.

In addition, we added reported latrine use to the first paragraph of Results to ensure the information is available before the Discussion:

“During the same visit, **100% of index households in the sanitation arm reported primarily using a latrine in their compound for defecation, compared to 97% of controls**, and 86% of sanitation index households and 77% of controls reported that adults always used a latrine for defecation.”

-       Lines 314 - 319: The onset of the substudy occurred 1 year post-intervention. This means intervention sites would have been subject to 1 year of "intensive promotion" prior to collection of the data referenced in lines 314 - 316? It would be more informative to include latrine use habit prior to intervention implementation.

We have added available latrine ownership information from the baseline of the parent trial (54% in either arm). We also added text that, while the latrine use practices measured in the sanitation arm reflect the influence of the intervention after one year of intense promotion, the practices among controls who received no promotion likely reflect baseline conditions (new text in bold):

“At the onset of this substudy, **approximately one year after intervention implementation in the sanitation arm,** 97% of index households in the control arm **reported using a latrine within their compound** (vs. 100% of index households in the sanitation arm,) and 77% reported that adults used the latrine exclusively for defecation (vs. 86% in the sanitation arm**). As the control arm did not receive any interventions or behavior change promotion, we expect the conditions among controls to represent baseline conditions before the onset of the parent trial. Only 54% of index households in either arm owned their own latrine at baseline of the parent trial.[16]”**

This clarification further led us to expand on our discussion of the intervention’s effects. In addition to the role of latrine upgrades, we included more discussion on how increased access to a personal latrine among index households, along with latrine upgrades, may have enabled the observed effects. New text is in bold, and removed text is not indicated.

“… **Providing a new, personal latrine to any index household that did not have one may have helped increase latrine use in the sanitation arm by reducing barriers associated with intra-compound latrine sharing, such as privacy or availability concerns.** **However,** latrine use habits appear to have been in place in the community even before promotion, and existing habits and norms may have facilitated sustained maintenance and use of latrines beyond the period of intensive promotion.

In addition, the sanitation intervention **included compound-wide** improvements in latrine quality. **This included latrine upgrades among index households that already owned a latrine at baseline and upgrades to any other existing latrines in the compound.** Compared to controls, latrines **primarily used by index households** in the sanitation arm were more likely to be pour-flush (99% vs. 76%) and have a functional water seal (95% vs. 46%), and less likely to be shared with other households (20% vs. 54%). Our findings therefore indicate that, in a setting with existing latrine use habits **and prevalent latrine sharing, provision of personal latrines** and improvements to latrine quality achieved a sustained reduction in child diarrhea.”

-       Line 319: The "backdrop" described is 1-year post-intervention as far I understood from the study design, so this sentence is confusing. To make this point, it would be useful to include data from the baseline phase of the parent study if available.

This text has been changed as described in response to your previous comment. “Against this backdrop…” was removed.

-       Lines 321 - 323: Clarify the unit of measurement - is this all existing latrines in a compound? Just latrines for index households?

Latrines for index households. Text corrected to: “Compared to controls, latrines **primarily used by index households** in the sanitation arm were…”

-       Lines 323 - 324: Again, I'm not sure this follows if the reference point for "a setting with existing latrine access and latrine use" is 1 year post-intervention (see first paragraph of results section where it is explained that the data are taken 1-year post-intervention).

The text has been changed as described above. We do assume that latrine access and use is relatively stable over this time period in the control arm, so that latrine access in the control arm at baseline of this study likely reflects a similar situation one year prior. However, we captured other aspects of sanitation access in the parent trial baseline survey and we have added this information to the manuscript.

Noting the difference between access to a latrine within the compound vs. owning a latrine was still helpful in clarifying our findings and Discussion.

-       Lines 335: Is the age distribution between the two groups being compared similar (parent trial vs sub-study)? If not, this could be a potential reason for the discrepancy?

We compared the prevalence during overlapping periods to capture temporal changes as well as to ensure the age distribution was similar. We added to the sentence to highlight this more clearly:

“During the period when both studies were ongoing**, and the ages of children in both studies were similar,** the seven-day prevalence of diarrheal disease among controls was 16.0% in this substudy vs. 5.5% in the parent trial…”

-       Given the difference in promotional activities & diarrhea effects between the Kenya and Bangladesh sites, it seems odd that it is not referenced in the discussion of the role of intervention promotion.

Intervention uptake was generally lower at the Kenya site, and differences in intervention delivery and behavioral promotion activities might explain differences in effects on diarrhea. However, there are many alternative reasons that the results in Bangladesh could differ from results in Kenya. We prefer not to make comparisons between study sites without a thorough exploration of all the differences in regions, background sanitation norms, and local contexts through which behavioral promotion and infrastructure upgrades were enacted. These comparisons would be valuable but are beyond the scope of this paper.

Figure S1: Rather than follow-up #, include indication of months post intervention. Follow-up round number is not informative in this figure.

In addition to follow-up number, we added the median time (in years) post intervention implementation. These are approximations due to the rolling nature of intervention implementation.

We changed the figure label to: “**Figure S1**: Flow chart of study participants in sanitation (left) and control (right) arms over each survey round. Survey rounds labeled with the median approximate time passed since intervention implementation.”

***** Reviewer #3:**

General Comments

This manuscript presents a robust analysis of the longitudinal effects of a sanitation intervention on child diarrhea and respiratory infections. The finding of a persistent reduction in diarrhea prevalence more than three years after the initial delivery of the intervention is well supported and provides confirmatory evidence for the shorter-term effect reported previously from the parent study. A truly interesting, and unexpected, observation arising from this sub-study is the substantially and consistently higher baseline risk for all caregiver-reported outcomes (including the negative control) compared with the parent study, which the authors explore in commendable depth. The description of these two parallel, partially overlapping assessments of the same outcomes using similar instruments is a valuable contribution in its own right, illustrating important discrepancies and raising critical questions relevant to the design and analysis of complex environmental health interventions in real world settings.

Thank you for your review and comments. We agree that the difference in prevalence estimates between overlapping assessments is an interesting result that is important for any research using caregiver- or self-reported symptoms.

Specific Comments

L69: Missing a verb in "when latrine use practices in place"

Changed to “are in place”.

L204: Please state how prevalence estimates and CIs were computed; the captions to Figures 1, 2, and S3-S5 mention "credible intervals", which would suggest some sort of Bayesian approach was used.

Updated our descriptive methods section to clarify (new text in bold):

“We estimated the prevalence of diarrheal disease and ARI during each month of follow-up separately for the sanitation and control groups to observe temporal and seasonal changes. **We estimated Bayesian credible intervals to account for sampling error, which used a beta distribution to restrict proportion estimates to values greater than zero.**”

…

“To investigate temporal variability and the impact of child age, we compared the monthly prevalence of diarrheal disease during overlapping periods when both studies were active, **using credible intervals to account for sampling error**.”

For model results elsewhere, standard confidence intervals were fit using generalized linear models.

L228 - 232: Was any effort made to differentiate between promotion intensity and child age? As I understand the study design, the low/no promotion intensity period would coincide with children being a year or two older, on average, than they were during the high promotion intensity period.

The reviewer is correct that the birth cohort is older during the low/no promotion period than during the high promotion period (from Results, second paragraph: “The median age of children at the first and last visits was approximately 15 and 38 months…”.

Therefore, if there were heterogeneity in intervention effects by child age, this could influence the magnitude of intervention effects detected in the two periods. In response to this comment, we assessed child age (continuous variable) as an effect modifier. The interaction term was not statistically significant (p = .7). This was not a formal analysis, but it does provide additional support for the interpretation of our existing results.

Table 2: It is helpful to see EMM assessed on both multiplicative and additive scales—well done! However, why is the dry-season diarrhea PD positive when the raw prevalence estimates and the PR all indicate lower prevalence in the sanitation arm during the dry season?

This was an error and has been corrected. Thank you for noticing.

L338-340: Were the parent study and sub-study data collection visits ever performed concurrently or within the same 7-day period?

Out of 9,088 observations in the substudy, 64 took place within 7 days of a parent trial survey. There are some children reported to have diarrhea during one survey but not the other, but almost all agree. This is a rare outcome among a small subset of observations, so it is difficult to glean anything from these observations. Discrepancies do not necessarily indicate misreporting, since one survey could take place within 7 days of the diarrheal episode but not the other survey.

This was an interesting exercise and well worth checking.

L350-351: This interpretation is compelling. However, can any arguments be made in favor of the parent study providing more accurate measurement of these caregiver-reported outcomes?

We do not find any compelling reasons that the parent trial’s measurements would be more accurate, but we acknowledge that the self-reported symptoms in either study could be biased. The original prevalence estimates in the parent trial were notably lower than anticipated, which generated substantial discussion.

Figure S2: Are the observation counts for the parent study across all children (including those not enrolled in this sub-study), or among sub-study children only?

Added “Parent trial data includes substudy participants and those not enrolled in the substudy.” to the figure caption.
