# Supplementary material for: Evaluation of an on-site sanitation intervention against childhood diarrhea and acute respiratory infection 1 to 3.5 years after implementation: Extended follow-up of a cluster-randomized controlled trial in rural Bangladesh
Source: PLoS Med. 2022 Aug 8;19(8):e1004041. doi: 10.1371/journal.pmed.1004041 (PMC9394830; doi:10.1371/journal.pmed.1004041)
Supplement: Attachment — Submitted filename: WASHB-longitudinal_child_health_response_May-28-2022.docx [file pmed.1004041.s007.docx]

**Evaluation of an on-site sanitation intervention against childhood diarrhea and acute respiratory infection 1 to 3.5 years after implementation: Extended follow-up of a cluster randomized controlled trial in rural Bangladesh**

Please finalize the arrangements for data deposition.

Data are now available at the link specified in our Data Availability statement (<https://osf.io/6u7cn/>).

At line 38, we suggest "... composite sanitation intervention" or similar.

Changed to “multi-component sanitation intervention”.

Again at line 38, we ask you to soften the wording slightly, to "... led to a reduction of diarrhea by 39% and of ARI by ...", say. Please make similar changes around line 141.

At line 38, changed to: “led to a 39% reduction in the prevalence of diarrhea among children and a 25% reduction for ARI, measured 1-2 years after intervention implementation.”

At line 141, changed to: The Maputo Sanitation (MapSan) controlled before-and-after study … found no change in child diarrhea”.

At line 145, changed to: “In Bangladesh, the sanitation intervention led to prevalence reductions of 39% for diarrheal disease, 25% for Giardia infections, 29% for Trichuris trichiura infections, and 25% for ARI.”

At line 52 and elsewhere, please use the style "... 1 year of ..." although numbers should be spelt out at the start of sentences.

Changed numbers to numerical form in all cases.

Changed one to 1 or “a single”, where more appropriate, and rephrased some sentences to remove need for numbers.

- Line 221: “The sanitation intervention included double-pit pour flush improved latrines, a sani-scoop for the removal of child and animal feces, and a children’s potty, along with in-person behavioral promotion on product use and maintenance”. [Removed “3 products”]
- Line 331: “We assessed potential effect modification by living in index vs. non-index households in the compound, periods of high-intensity promotion vs. low-intensity or no promotion, and monsoon vs dry. Season.” [Removed “3 potential effect modifiers”]

At line 60, is there a way to avoid "0.000"?

Changed 0.000 to 0 in the abstract and text, but left as is in the results tables.

At line 68, we ask you to adapt the text to "... appeared to be sustained ..." or similar, considering the statistical evidence seen.

Added “appeared to be”, as suggested.

At line 90 (author summary) and any other instances, please make that "... under 5 years".

All instances changed to “… under 5 years”.

At line 159, please make that "... we used".

Corrected.

Should the numbers at line 296-7 not add up to 9,088?

The total number of observations was incorrectly recorded. The correct total was 9,800. We corrected the text in two places.

At line 349, please remove "by 19%" (which is implicit in the quoted PR). 

Removed “by 19%” and moved “from 14.5% to 11.9%” before the PR in parentheses.

Given the observed p value, please also soften the wording here to "... was associated with a reduction in the prevalence of ...".

Changed as suggested.

Throughout the text, please ensure that reference call-outs precede punctuation (e.g., "... among children [4].").

Corrected throughout.

Please finalize the citation for reference 27. Prior to publication of the present paper, this reference will either need to be available in published form, made available as a preprint, or removed.

This manuscript is still under review. We elected to replace the reference with a similar study conducted using the full parent trial data. Results were similar, and the new reference supports the claim we made.

“Child potties and sani-scoops did not likely contribute to intervention effects, as use of these products was low and inconsistent and child feces management practices remained poor in the sanitation arm [27].”

New reference:

27. Parvez SM, Azad R, Rahman M, Unicomb L, Ram PK, Naser AM, et al. Achieving optimal technology and behavioral uptake of single and combined interventions of water, sanitation hygiene and nutrition, in an efficacy trial (WASH benefits) in rural Bangladesh. Trials. 2018;19: 1–16. doi:10.1186/s13063-018-2710-8

Is reference 29 missing a journal or source name?

The source was missing. Reference updated:

Zaman Y. Machine Learning Model on Rainfall-A Predicted Approach for Bangladesh. UIU Institutional Repository [Master's thesis, United International University, Dhaka, Bangladesh]. 2018. Available at: http://dspace.uiu.ac.bd/handle/52243/178

Please rename the attached CONSORT checklist "S1_CONSORT_Checklist".

Changed.

Thank you for including the Islam et al draft paper, and this can now be removed.

Will remove from submission.

Move supplementary figure 1 into the main ms (i.e., convert it to figure 1)? Sorry to have neglected to include this in our earlier letter.

Changed, and all figures re-numbered. New supplement figures uploaded with updated filenames.

Comments from Reviewers:

*** Reviewer #1:

Thank you to the authors for addressing all my previous comments well. I have no further issues to raise.

*** Reviewer #2:

I am satisfied with the author responses to my comments and thank the authors for their thoughtful and comprehensive responses. I enjoyed reading and reviewing the paper.

*** Reviewer #3:

The authors have satisfactorily addressed the previous reviewer comments.
